# *Quercus wutaishanica* shrub affects temperate forest community composition and soil properties under different restoration stage

**DOI:** 10.1371/journal.pone.0294159

**Published:** 2023-11-17

**Authors:** Peng Kang, Jiming Cheng, Jinpeng Hu, Yongshun Jing, Jing Wang, Hui Yang, Xiaodong Ding, Xingfu Yan

**Affiliations:** 1 College of Biological Sciences and Engineering, North Minzu University, Yinchuan, China; 2 Key Laboratory of Ecological Protection of Agro-Pastoral Ecotones in the Yellow River Basin, National Ethnic Affairs Commission, Yinchuan, China; 3 Forest Tree Breeding Center, Liupanshan Forestry Bureau, Guyuan, China; Qingdao Agricultural University, CHINA

## Abstract

*Quercus wutaishanica* is the dominant tree species in the natural ecosystem restoration of temperate forests in China, and it plays an active role in maintaining ecological balance. However, little is known about how ecosystem versatility develops during the restoration of forest ecosystems dominated by *Q*. *wutaishanica*. In this study, we investigated the species composition of *the Q*. *wutaishanica* community, soil nutrients, and their functional traits at various restoration stages, and comprehensively analyzed the correlations among them. At the early stage of restoration (10 years of restoration), there were *Spiraea pubescens* and *Syringa pubescens* in *Q*. *wutaishanica* community (87% of the total species), while had a larger niche width. In the middle of restoration (30 years of restoration), shannon and evenness indices were the largest, while soil total carbon, ammonium nitrogen and chlorophyll content of *Q*. *wutaishanica* leaves were the highest; among them, soil total carbon was 15.7% higher than that in 10 years of restoration, 32.4% higher than that in 40 years of restoration, ammonium nitrogen was 71.7% higher than that in 40 years of restoration, and chlorophyll content was 217.9% higher than that in 10 years of restoration, and 51.8% higher than that in 40 years of restoration. At the later stage of restoration (40 years of restoration), *Lonicera ferdinandii* occupied the dominant ecological niche, and soil available nitrogen, available phosphorus content and leaf thickness were the largest; while AN was 10.9% higher than that of 10 years of restoration, 16.5% higher than that of 30 years of restoration, AP was 60.6% higher than that of 10 years of restoration, 21.6% higher than that of 30 years of restoration, leaf thickness was 22.3% higher than that of 10 years of restoration, 84.9% higher than that of 30 years of restoration. However, the restriction of various soil nutrients was reduced. Our study highlighted the effectiveness of soil resource availability in plant communities during restoration, reduced competition for light among plants, and altered species richness. Furthermore, changes in the interrelationship between plant community composition and leaf functional traits of the dominant species responded positively to community restoration. These results further deepen our understanding of forest management and restoration of forest communities. In the future, it is necessary to comprehensively consider the influence of various factors on forest community restoration.

## 1 Introduction

The Grain to Green Program (GTGP), Natural Forest Protection Program (NFPP), and Three-North Shelterbelt Program (TNSP) are a series of important plans for the restoration of natural ecosystems in China, which significantly impacts the maintenance of ecosystem functions, biological diversity, and carbon balance [1–3]. With the implementation of ecological restoration plans, most evergreen broad-leaved forests are currently at different restoration stages after being restored to secondary forests [4, 5]. *Quercus wutaishanica* is the dominant tree species in warm and temperate deciduous broad-leaved forests and mixed broadleaf-conifer forests in China and plays a positive role in soil and water conservation and ecological balance maintenance [6]. Therefore, improving our understanding of the changes in plant diversity, soil properties, and plant functional traits during ecosystem restoration is essential for coping with abiotic and biotic stresses (climate change, insect pests, drought, etc.) and for the proper management of forests.

During forest ecosystem restoration, plant diversity has been studied as an indicator of community species composition and diversity [7]. Long-lived tree species can reduce the fragmentation of the forest landscape at the spatiotemporal scale [8], and tree species with larger canopy gaps are conducive to the replenishment of early stages [9]. With restoration, the number of dominant species and pioneer species has increased [10, 11]. Therefore, the study of plant diversity at various stages of restoration can provide a theoretical basis for the restoration and reconstruction of forest ecosystems. However, unlike the traditional use of plant diversity in assessing the restoration stage of forest ecosystems, various aspects of nutrient accumulation in forest ecosystems have been developed in recent studies [12, 13]. An increase in forest carbon sink can improve the diversity and stability of plant community structure [14, 15], while nutrient inputs (e.g., nitrogen or phosphorus) have significant negative effects on plant community richness, thus increasing plant community similarity and decreasing species diversity [16].

An increasing number of studies have shown that in addition to disturbances, environmental conditions (soil properties) also one of the factors affect species diversity during the restoration stage [17]. Soil nutrients, as important regulators of vegetation regeneration, exhibit different characteristics at different forest restoration stages, and are closely related to forest restoration speed [18]. The establishment of woody plants is delayed by the coverage of annual plants when nutrients are abundant [19]. With the depletion of soil nutrient resources, plants with slow growth rates have more advantages than those with fast growth and high reproductive rates [20]. In addition, the accumulation of soil nutrients depends on the vegetation composition. During the restoration stages, soil total phosphorus content decreased gradually, whereas total nitrogen content and alkali-hydrolyzable nitrogen in organic matter increased significantly [21]. Other studies have indicated that soil carbon, nitrogen, and phosphorus contents first decreased and then increased with restoration, and were higher during the late restoration periods [22]. More studies have suggested that with an increase in tree biomass, soil nutrients, organic matter, and carbon storage also increase [1]. In conclusion, the differences between the dominant species and the number of species in the forest restoration stage are closely related to soil nutrients [23]. Soil nutrients, including exchangeable cations, total nitrogen, and phosphorus, have strong geographical heterogeneity and affect the composition of plant species at different restoration stages as well as species functional traits [24].

In recent years, studies on plant functional traits have greatly improved our understanding of plant functions and characteristics during forest restoration [25, 26]. Previous studies have shown that the ecological strategies of plants switch between resource acquisition and conservation during the restoration stage [27]. However, plants must make trade-offs between leaf physiological traits and nutrient storage to better adapt to biotic and abiotic factors during restoration [28]. From dry to wet conditions, plants use more water to maintain their growth while decreasing photosynthesis to increase protein consumption in leaves with lower nitrogen content [29]. At the early stage of restoration, herbaceous plants dominate, and woody plants improve their photosynthetic capacity by accumulating nitrogen in leaves to maintain dominant growth [30]. Overall, biomass allocation among plant organs is driven by environmental conditions, but functional traits may also be potential variables for biomass allocation [31].

Previous studies have suggested that the dominant geographical boundary factors (climate and disturbance intensity) affect the distribution of *Q*. *wutaishanica* in China [32], which focused on the relationship between spatial distribution patterns, plant functional traits, nutrients, soil carbon, nitrogen, and phosphorus in *Q*. *wutaishanica* communities in the Taiyue and Ziwuling Mountains [33–36]. Therefore, we proposed the following hypotheses: (1) there would be differences in species diversity and richness within communities at different restoration stages of *Q*. *wutaishanica* communities; (2) changes in species composition during the restoration stage of *Q*. *wutaishanica* communities may also have an effect on soil characteristics; and (3) changes in leaf functional traits of dominant plants in *Q*. *wutaishanica* communities would respond differently to the restoration stage.

## 2 Materials and methods

### 2.1 Study site

**Fig 1:**
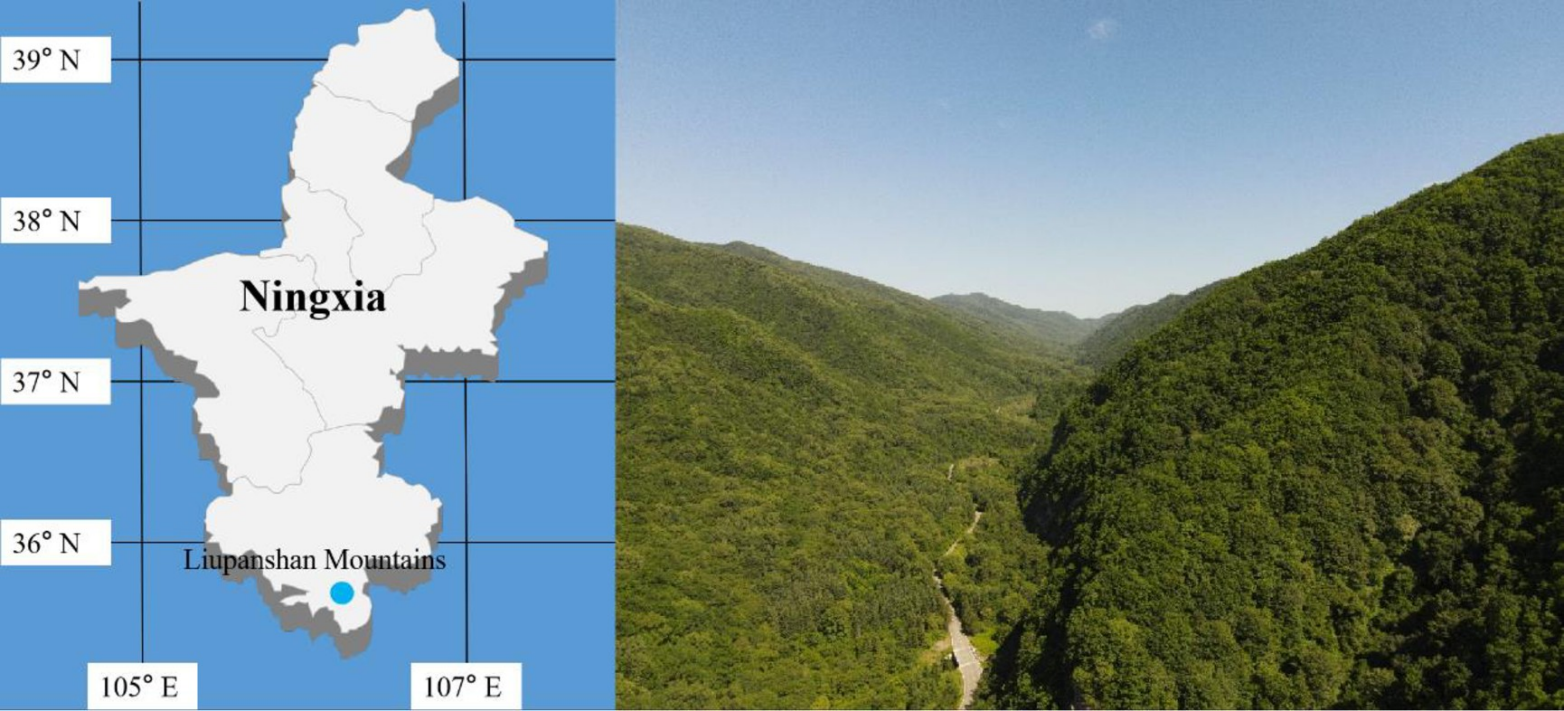
Distribution of sampling sites in Liupanshan Mountains of Ningxia, China.

This study was conducted in the National Nature Reserve of the Liupanshan Mountains (35°15′ to 35°41′ N and 106°09′ to 106°30′ E), which is located in the south of the Liupanshan Mountains in Guyuan City (Longde and Jingyuan counties) of the Ningxia Autonomous Region, with a total area of 67,800 ha, and is one of the largest forest areas in northwest China (Fig 1). And we obtained the approval of Forest Tree Breeding Center, Liupanshan Forestry Bureau, Guyuan Fig 1 from https://apps.nationalmap.gov/viewer/.

The National Nature Reserve of the Liupanshan Mountains is located at the edge of the northern agriculture/pastoral ecotone and a semi-humid to semi-arid transitional zone in North China. Cold and dry air flows were interlaced. It is hot and rainy in the summer and dry and cold in the winter. The mean annual temperature is 5.8°C, the coldest month (January) is −7°C, and the hottest month (July) is 17.4°C. The mean annual precipitation is 676 mm, and the mean annual evaporation is approximately 1426 mm. The frost-free period was approximately 120 days, and the annual total sunshine duration was 2100–2400 h. There are more than 80 rivers in the Liupanshan Mountains, all of which belong to the Yellow River System. The soils in the area are grayish brown.

Through visits to the Forest Tree Breeding Center, Liupanshan Forestry Bureau, Guyuan, and combined with information detailed by local villagers, we selected areas that were completely destroyed before restoration, where the shrubs were cut down and the dominant tree species were restored naturally. In July 2019, we identified three shady slope sample stands that gradually extended from east to west, from near the Yejia village in Baimian Town to Liupanshan National Nature Reserve. The first sample stand was Xilianggou (E106°21′, N35°23′), which was restored late and only gradually with the recent reduction of human activities such as logging and grazing, and the restoration time is about 10 years from now (10 y). And the elevation was about 1849.13 m. The second sample stand was Dawan (E106°21′, N35°23′), which was fenced and restored in the 1990s, and the restoration time was about 30 years from now (30 y). The elevation was about 1882.13 m. The third sample stand was Dadaogou (E106°21′, N35°23′), located in the Liupanshan Nature Reserve, which was fenced and restored in the late 1970s, about 40 years ago (40 y). And the elevation was about 1912.98 m (Table 1).

**Table 1:** Restoration time, latitude, longitude, and altitude of the three study sites in the Liupanshan Mountains.

Study site	Restoration time	Latitude and longitude	Altitude (m)
Xilianggou	10 years	35°23′N, 106°21′E	1849.13
Dawan	30 years	35°23′N, 106°21′E	1882.13
Dadaogou	40 years	35°23′N, 106°21′E	1912.98

The main vegetation types in the Liupanshan Mountains are temperate coniferous forests, evergreen Bambusoideae shrubs, secondary deciduous broad-leaved forests, deciduous broad-leaved shrubs, and grasslands. The temperate coniferous forest is dominated by *Pinus armandii*, the evergreen bambusoideae shrub by *Fargesia nitida*, and the secondary deciduous broad-leaved forest by *Q*. *wutaishanica*, *Betula platyphylla*, *Betula albosinensis* and *Populus davidiana* [37].

### 2.2 Community investigation and species composition index calculation at various restoration stages

We investigated the composition of plant communities in three sample stands and determined soil physicochemical properties and plant functional traits. Four transect lines with the same slope direction were set in each stand, and the spacing of each line was greater than 20 m. A 10 ×10 m quadrate was randomly set on each line transect using a simple random method. Each sample stand has 4 quadrats, for a total of 12 quadrats. All woody plants with a base diameter ≥1 cm in each quadrate were counted, and their species names, plant height, plant number, and coverage were recorded. The plant species were identified with the method of Cheng et al. (2020) and combined with data from http://ppbc.iplant.cn/ [38].

Species diversity indices, including shannon index [39], evenness index, and richness index [40, 41], were calculated according to the following equations:

shannon index: (1)
evenness index: (2)
richness index: (3)

where *P*_*i*_ = n_i_/N, n_i_ represents the number of individuals of a species, N is the total number of species, and S is the number of species.

Niche width was calculated using the shannon formula [42] and niche overlap was calculated using the Pianka index [43] (Fig 3).

shannon index: (4)
Pianka index: (5)

In the formula, *B*_*i*_ is the niche width of i species; *P*_*ij*_ = n_ij_/ N_i_, n_ij_ represents the importance value of i species in j resources, N_i_ is the sum of the importance values of i species in total resources, *P*_*ij*_ is the proportion of the importance value of i species in j resources to that species in total resources, r the number of quadrats, and *O*_*ik*_ is the overlap index of species i and k.

### 2.3 Analysis of soil nutrients at various restoration stages

Soil samples were collected from three sample stands at various restoration stages with consistent environmental and terrain conditions. In each 10 × 10 quadrate, nine sampling points were selected according to the S-shape sampling method, and the soil from each of the three sampling points was mixed into one soil sample, while soil mixtures with a depth of 0–10 cm were collected with a shovel. Twelve soil samples were collected at three different restoration stages, and the collected soil samples were placed in valve bags and brought back to the laboratory. Each soil sample was divided into two parts. One part was screened a 0.25 mm sieve and then dried naturally. After grinding, the total organic carbon (TC, g/kg), total nitrogen (TN, g/kg), total phosphorus (TP, g/kg), available phosphorus (AP, mg/kg), and available potassium (AK, mg/kg) content were measured [44]. Another portion of fresh soil from each sample was screened using a 2 mm sieve and stored at 4°C for the measurements of soil ammonium nitrogen (NH_4_^+^-N, mg/kg), nitrate nitrogen (NO_3_^—^N, mg/kg), and available nitrogen (AN, mg/kg) contents [45].

### 2.4 Analysis of plant functional traits of *Q*. *wutaishanica*

We collected the upper canopy and sunny, pest-free, and fully unfolded leaves of *Q*. *wutaishanica* plants from each stand in September 2019. All leaves were placed between two pieces of wet filter paper, placed in a valve bag, and brought to the laboratory for measurement [46]. The leaves were divided into two parts on average. One part was used to measure plant functional traits: leaf length-width ratio (LWR), leaf thickness (LT, mm), leaf dry matter content (LDMC, g), specific leaf area (SLA, cm^2^/g), and leaf chlorophyll content (LCC, mg/g). Leaf length was measured as the distance from the petiole-blade junction to the blade tip, and leaf width was measured at the widest part of the blade. A digimatic micrometer was used to measure three positions (front, middle, and back) of the blade close to the main veins, and LT was the average value obtained from the three measurements. Furthermore, leaves were soaked in a petri dish filled with water under 5°C dark conditions for 12 h, the water on the surface was quickly wiped off with filter paper, and the saturated fresh weight of the leaves was weighed immediately. The leaves were then dried in an oven at 75°C to a constant weight and their dry weights were measured. LDMC was calculated as leaf dry weight divided by leaf saturated fresh weight. The leaf area was measured using a por. leaf area meter (LI-COR 3000C Area Meter, LI-COR, Lincoln, USA), and SLA was calculated as leaf area divided by leaf dry weight [47]. Chlorophyll in fresh leaves was extracted using acetone, and the chlorophyll content was measured using spectrophotometry [48]. The remaining leaf samples were placed in an oven at 80°C for 30 min and then at 60°C for 72 h. After complete drying, the samples were crushed and sieved through a 0.25 mm. These leaf samples were used to measure the total carbon (LTC, g/kg), total nitrogen (LTN, g/kg), and total phosphorus (LTP, g/kg), according to previous studies [44].

### 2.5 Statistical analysis

Soil nutrients and plant functional traits were analyzed by one-way analysis of variance (ANOVA) using SPSS (25.0), and multiple comparisons were performed for all data using the least significant difference method (LSD). All data are presented as mean ± SE (*n* = 12). The "Vegan" package was used in R software for non-metric multidimensional scaling (NMDS) analysis [49]. “Spaa” package in R software (Version 4.1.0) was used to calculate niche overlap based on levins method, and the corrplot package was used for visualization. The functional richness index (FRic), functional evenness index (FEve), functional divergence index (FDiv), functional dispersion index (FDis) were calculated by “FD” package. The correlations between soil physicochemical properties and plant functional traits and between plant functional traits were calculated using the "psych" package. Redundancy analysis (RDA) was used to establish relationships among species composition, soil physicochemical properties, and plant functional traits at various restoration stages [50].

## 3 Results

### 3.1 Species composition of *Q*. *wutaishanica* community among various restoration stages

**Fig 2:**
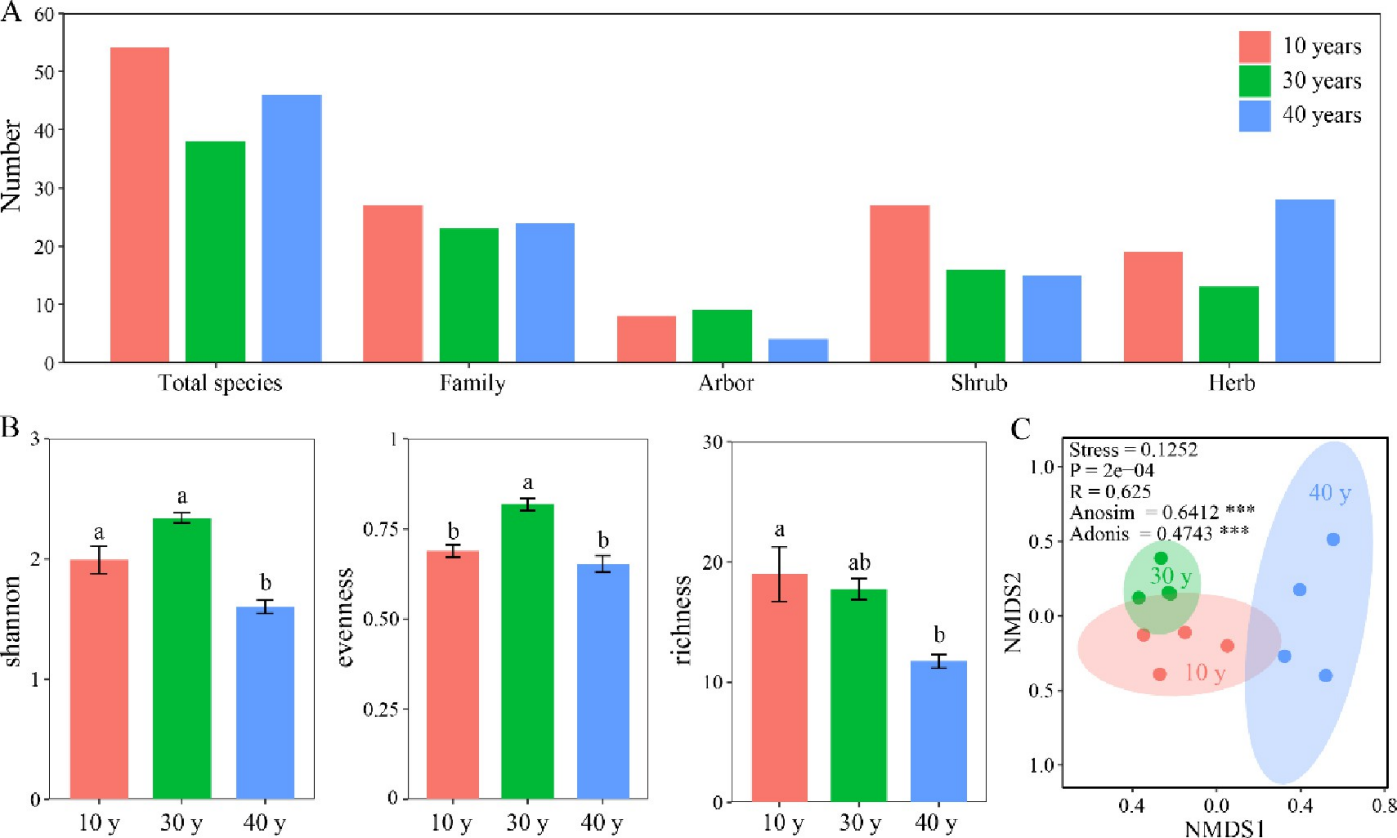
Species composition characteristics (A), the indices of species diversity (B) and non-metric multidimensional scaling analysis (C) of *Quercus wutaishanica* shrub at various restoration stages in Liupanshan Mountains.

By surveying all species in three different restoration stages, we found 10 species of arbors, 31 species of shrubs, and the most herbaceous species with 42. Among the three different restoration stages, 30 years of restoration contained the most trees with nine species, 10 years of restoration was the second with eight species, and 40 years of restoration had the fewest tree species with four. The number of shrubs after 10 years of restoration was the largest, with 27 species, accounting for 87% of the total; 30 years of restoration was the next most abundant with 16 species, accounting for 51% of the total; and 40 years of restoration was the least abundant with 15 species, accounting for 48% of the total (Fig 2A).

At the three restoration stages, shannon and evenness indices were the largest at 30 years of restoration, which were significantly different from those at 40 years of restoration (*p* < 0.05). The richness index was the largest at 10 years of restoration and significantly higher than that at 40 years (*p* < 0.05) (Fig 2B). NMDS analysis showed that plant communities were similar after 10, 30, and 40 years of restoration (Fig 2C).

**Fig 3:**
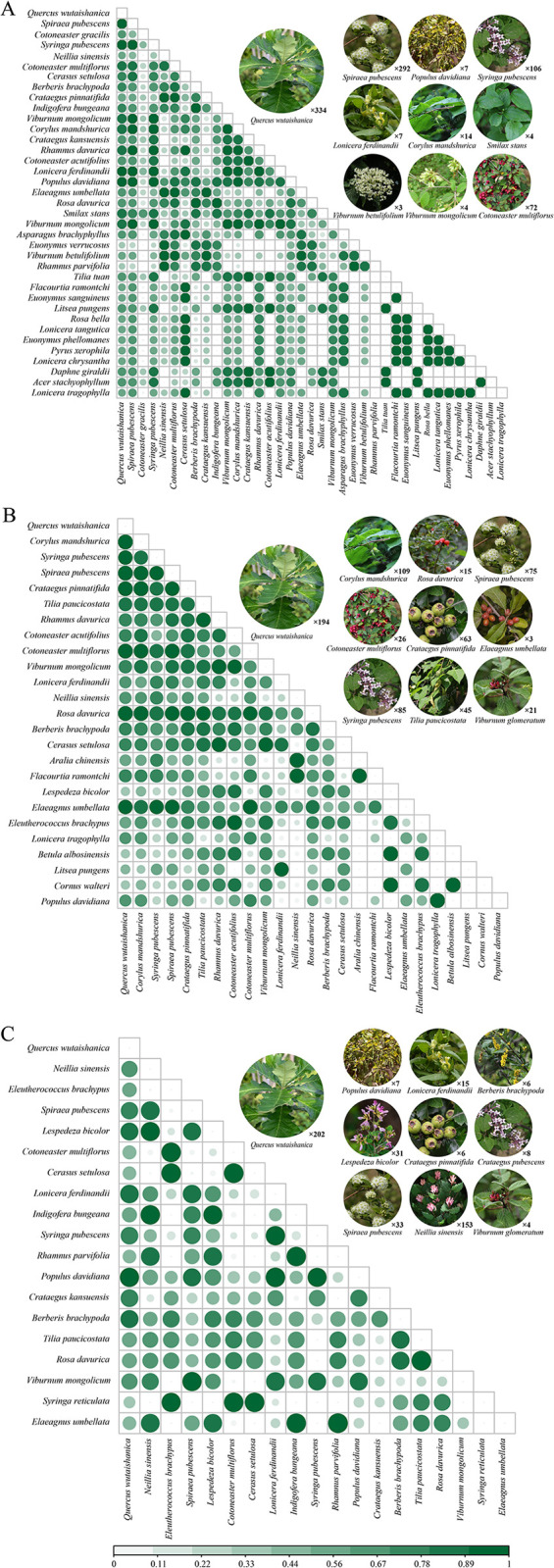
Niche overlap of shrubs in *Quercus wutaishanica* shrub at various restoration stages in Liupanshan Mountains. A: restoration for 10 years; B: restoration for 30 years; C: restoration for 40 years. The plants in the figure are the top 10 ecological niches.

At 10 years of restoration, *Q*. *wutaishanica* had a higher niche overlap with *Spiraea Pubescens*, *Populus Davidiana* and *Syringa Pubescens* (Fig 3A). At 30 years of restoration stage, *Q*. *wutaishanica* had a higher niche overlap with *Corylus mandshurica*, *Rosa davurica* and *Spiraea pubescens* (Fig 3B). At 40 years of restoration stage, *Q*. *wutaishanica* had a higher niche overlap with *Populus davidiana*, *L*. *ferdinandii* and *Berberis brachypoda* (Fig 3C).

### 3.2 Characteristics of soil nutrients at various restoration stages

**Fig 4:**
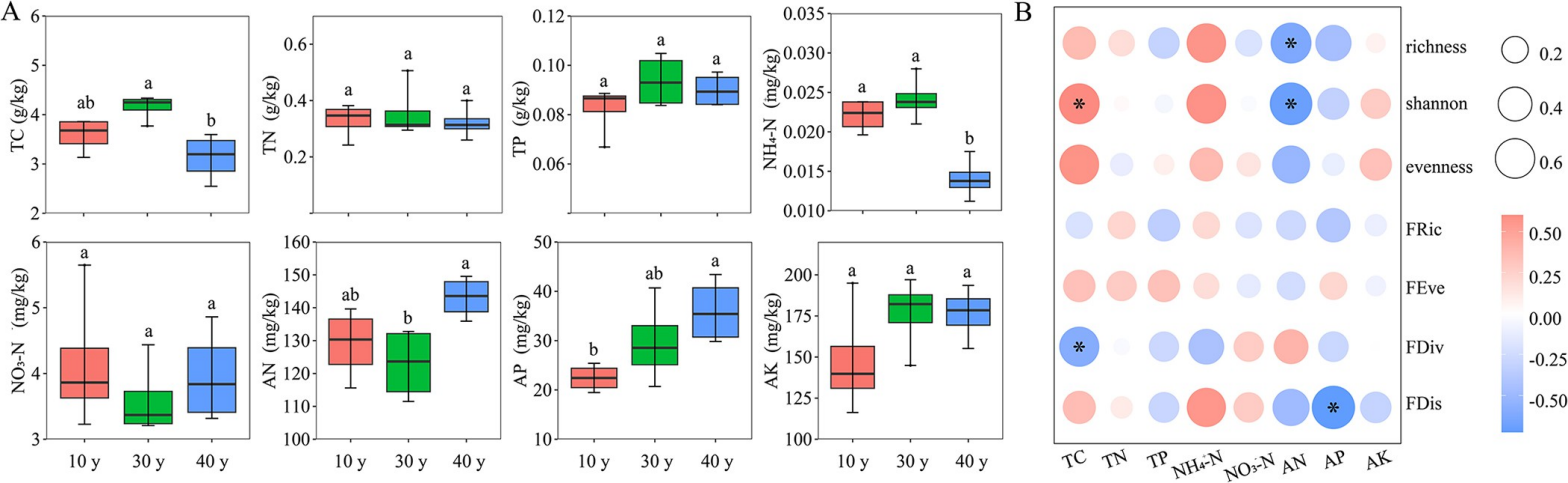
Relationship between soil physicochemical properties (A) and community diversity index (B) at various restoration stages in Liupanshan Mountains. TC: total carbon; TN: total nitrogen; TP: total phosphorus; NH_4_^+^-N: ammoniacal nitrogen; NO_3_^—^N: nitrate nitrogen; AN: available nitrogen; AP: available phosphorus; AK: available potassium; FRic: functional richness index; FEve: functional evenness index; FDiv: functional divergence index; FDis: functional dispersion index. (*p < 0.05; **p < 0.01).

The soil TC content was highest at 30 years of restoration and significantly higher than that at 40 years of restoration (*p* < 0.05). The NH_4_^+^-N content after 30 years of restoration was higher than that after 40 years of restoration. However, the AN and AP contents in soil after 40 years of restoration were significantly higher than those after 10 and 30 years of restoration (*p* < 0.05) (Fig 4A). Furthermore, the correlation analysis between the community structure characteristic index and physicochemical properties showed that the richness index of the plant community was significantly negatively correlated with AN, while shannon was positively correlated with TC, but negatively correlated with AN. In addition, FDis and RaoQ scores were negatively correlated with AP (*p* < 0.05) (Fig 4B).

### 3.3 Plant functional traits of *Q*. *wutaishanica* at various restoration stages

**Fig 5:**
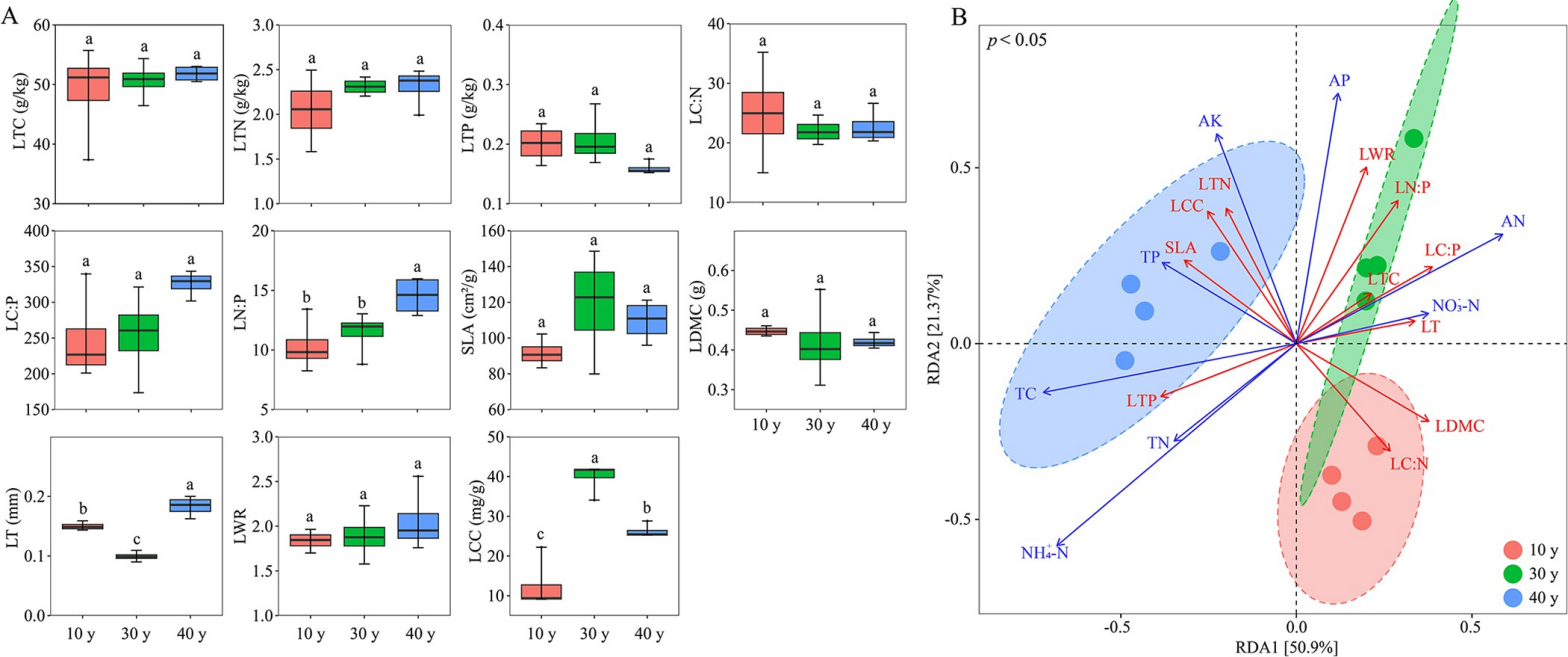
Plant functional traits (A) and RDA analysis (B) of *Quercus wutaishanica* shrub at various restoration stages in Liupanshan Mountains. LTC: leaf total carbon; LTN: leaf total nitrogen; LTP: leaf total phosphorus; LC:N: leaf carbon-nitrogen ratio; LC:P: leaf carbon-phosphorus ratio; LN:P: leaf nitrogen-phosphorus ratio; SLA: specific leaf area; LDMC: leaf dry matter content; LT: leaf thickness; LWR: leaf length-width ratio; LCC: leaf chlorophyll content.

Our study indicated that the LTC content of *Q*. *wutaishanica* leaves showed little difference between the three restoration stages. LTN content after 30 and 40 years of restoration was higher than that after 10 years, and LTP content decreased with restoration time. The LT index was the highest at 40 years of restoration and was lower at 30 years; LCC was the highest at 30 years of restoration and significantly higher than that at 10 and 40 years of restoration (*p* < 0.05) (Fig 5A). RDA showed that leaf functional traits and soil physicochemical properties had strong directivity to restoration years, while RDA1 and RDA2 explained 50.9% and 21.37% of the variables, respectively. Among them, LWR, LN:P, LTC, and LC:P were correlated with AP, AN, and NO_3_^—^N after 30 years of restoration, whereas LTP, SLA, LCC, and LTN were significantly correlated with soil TC, TP, and AK after 40 years of restoration (Fig 5B).

### 3.4 Relationship between community diversity index and plant functional traits of *Q*. *wutaishanica* community

**Fig 6:**
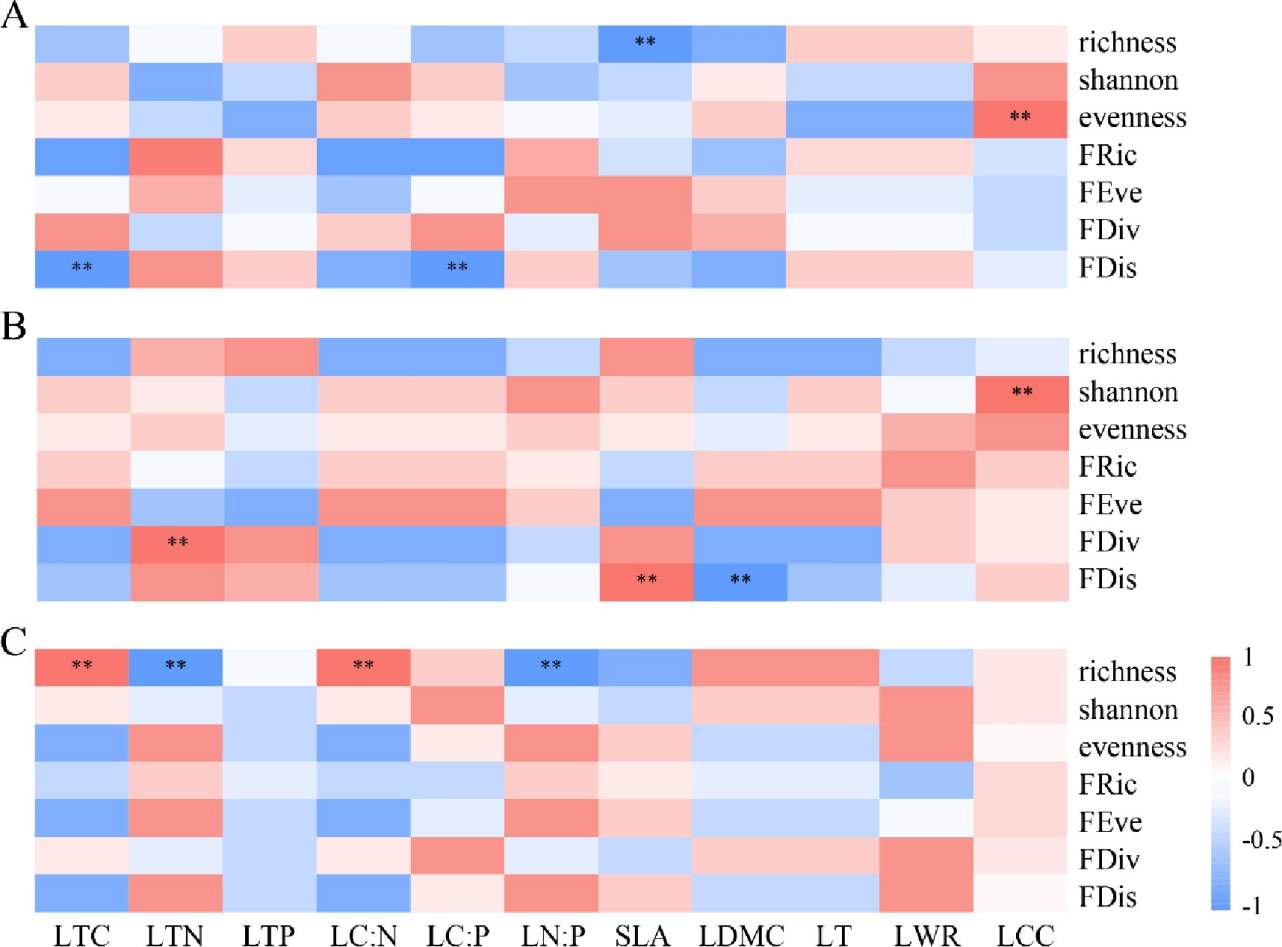
The spearman correlation with plant functional traits and community diversity index of *Quercus wutaishanica* shrub at various restoration stages in Liupanshan Mountains. A: restoration for 10 years; B: restoration for 30 years; C: restoration for 40 years. LTC: leaf total carbon; LTN: leaf total nitrogen; LTP: leaf total phosphorus; LC:N: leaf carbon-nitrogen ratio; LC:P: leaf carbon-phosphorus ratio; LN:P: leaf nitrogen-phosphorus ratio; SLA: specific leaf area; LDMC: leaf dry matter content; LT: leaf thickness; LWR: leaf length-width ratio; LCC: leaf chlorophyll content; FRic: functional richness index; FEve: functional evenness index; FDiv: functional divergence index; FDis: functional dispersion index. (*p < 0.05; **p < 0.01).

Further analysis showed that LTC and LC:P had a negative effect on FDis and RaoQ after 10 years of restoration, while LCC had a positive correlation with evenness (*p* < 0.05). With the restoration of plant communities, LTN, SLA, and LCC were positively correlated with the plant community indices (*p* < 0.05). At the 40 years of restoration, LTC and LC:N were positively correlated with richness, while LTN and LN:P were negatively correlated with richness (*p* < 0.05) (Fig 6).

## 4 Discussion

### 4.1 At the later restoration stages, the composition of the species community changes

*Q*. *wutaishanica* plays an important role in forest restoration in the Loess region of northern China [51]. Our study highlighted that the species diversity dominated by *Q*. *wutaishanica* communities changed with prolonged restoration time, which was consistent with the findings of Craven et al. [52]. In general, small-seeded species predominate in the early restoration of young (4–12 years) and middle (16–20 years) forests. However, in old-growth forests, there are some plants related to late restoration (such as large-seeded species and trees) [53]. After 10 years of restoration, the *Q*. *wutaishanica* community had more total species, which was similar to the result that plant species accumulated rapidly during the initial stage of restoration (Fig 2). Although environmental factors (sunlight, evaporation, and soil nutrients) have a significant impact on the distribution of plant species [54], ephemeral plants often dominate the community in the early restoration stage [55]. Abbas et al. also found that in the restoration of tropical forest plant communities, the accumulation of plant species mainly occurs in the early stage of restoration (15–20 years) [56]. With restoration, the number of shrubs in the middle of the restoration stage (30 y of restoration) decreased. This is similar to previous results showing that with the prolonging of the restoration period, the competition among species in the community intensified, leading to the decline of species [57, 58]. It is more likely that the community vegetation was dominated by sunny species in the early stage of restoration, and the species composition was differentiated in the middle stage due to random environmental filtering and dispersal restrictions of species colonization and recruitment [56]. Especially in forest community restoration, pioneer species dominate the secondary forest community structure [59].

An important question in forest restoration is whether the diversity of plant species has improved [58]. The species diversity of the *Q*. *wutaishanica* community increased significantly after 30 years of restoration compared to that after 10 years of restoration. It is well known that the species richness of immature secondary forests is higher than that of young secondary forest [60, 61]. Species heterogeneity and diversity changed, probably because of the stronger response of new community species to soil nutrients and environmental changes at the early stages of restoration, as well as the limitation of seed dispersal [57, 62]. Lasky et al. found that species diversity in tropical forests decreased with increasing restoration period (from 10 to 40 years) [63]. Similarly, it was found that after a long period of forest restoration (50 years), species diversity decreased significantly [64, 65]. Our study found that the species diversity of the *Q*. *wutaishanica* community decreased after 40 years of restoration compared to that after 30 years of restoration. This may be because of the existence of pioneer species in the early restoration of the *Q*. *wutaishanica* community, which recruited a large number of species, and the lack of abiotic or biotic filtration, resulting in an increase in species diversity. With restoration, the species colonization rate was higher and the mortality rate was lower in the understory, while some dominant species and herbs eventually dominated in the understory, thus changing species diversity [66]. In the present study, plant species diversity first increased and then decreased (Fig 2). This unimodal curve was probably due to communities constructed by random dispersal and species colonization during early restoration. In view of the closer relationship between species, the environment was suitable for their expansion, which maximized species diversity in the middle stage of restoration. However, at the later stages of restoration, related species were gradually eliminated due to environmental filtration, which reduced the species diversity in the community [56, 67].

Our observations indicated that the plant species richness of the *Q*. *wutaishanica* community decreased gradually from 10 to 40 years after restoration (Fig 2B). Studies have shown that species richness gradually stabilizes with community restoration. The former was gradually shaded by the crown canopy, whereas the latter gradually occupied the crown canopy with restoration [61], thus playing the role of filtering restrictions and reducing species richness [68]. In addition, a higher soil resource supply and light availability at the initial restoration stage of the plant community increased plant richness. However, as plant community restoration proceeds, the availability of soil resources decreases, reducing consumer competition for light and altering species richness [69]. Furthermore, competition among plant consumers, which increases intraspecific community height, also decreases interspecific SLA [69, 70]. Plant community responses to restoration drivers depend on the underlying characteristics of resident species that influence community diversity during restoration [69].

In the restoration of plant communities, the larger the niche width of the species, the higher the resource utilization efficiency of the species, and the stronger the adaptive capacity to the environment [71]. Our results indicated that the niche width of *Q*. *wutaishanica* was the largest among the three restoration stages, indicating that *Q*. *wutaishanica* was dominant in each stage. The niche width of community restoration dominated by *Q*. *wutaishanica* was controlled by environmental selection and random processes; however, random processes may play a large role in the early stage of restoration, while environmental selection plays a dominant role in the middle and late stage [67, 72]. In addition, niche overlap reflects the similarity in resource utilization among populations. We found that species niche overlap significantly decreased after 40 years of restoration, which was similar to the results of previous studies that symbiotic relationships among plants gradually weakened over time, and competition among species decreased during community restoration at a regional scale [71, 72].

### 4.2 At the later restoration stages, the limitation of soil nutrients decreased

Forest ecosystem restoration is determined by the soil environment, especially soil nutrient availability. However, plant growth rate and biomass accumulation respond rapidly to soil nutrients [73]. It is believed that species in the early stage of restoration compete with other species by increasing the utilization efficiency of light resources through nutrient accumulation [74]. The reason why TC and NH_4_^+^-N in our study were the largest after 30 years of restoration was also confirmed, while the competitive growth of species increased the utilization of NH_4_^+^-N by plants and carbon input in soil (such as litter) [75]. With the restoration of the *Q*. *wutaishanica* community, the dominant species had a niche advantage and the demand for AN content further increased (Fig 4). The competing species died successively, which changed the carbon input to the soil [76]. This may explain the strong correlation between TC and community diversity observed in this study.

The change in soil AN content was opposite to that of TC and TP contents in this study and was the lowest after 30 years of restoration. On one hand, species diversity increased the AN utilization by plants, resulting in nitrogen limitation of soil nutrients [77, 78]; on the other hand, leguminous plants (*Medicago lupulina* and *Lespedeza bicolor*) appeared in 10 and 40-year restoration communities, which increased the fixation of available nitrogen in soil [79]. Another study pointed out that soil ammonium and nitrate nitrogen play key roles in community β-diversity composition [80]. In our study, the carbon nitrogen ratio and NH_4_^+^ were higher in the community after 30 years of restoration than those after 40 years of restoration, indicating that the ability of soil nutrient mineralization and decomposition gradually decreased during community restoration, and similar results were recorded by Bauhus and Pare [81].

### 4.3 Species composition and leaf functional traits of *Q*. *wutaishanica* communities differ in different restoration stages

Plant functional traits are indicators of plant resource utilization strategies and are closely related to various restoration stages of the community. The analysis of plant functional traits can deepen our understanding of community restoration processes and community structure [82–85]. It was observed that the strategy of resource utilization in plants was changed from acquisitive type to conservative type during restoration [86]. Here, we found that the SLA and LCC indices initially increased and then decreased with restoration. In plant restoration, owing to differences in soil nutrient allocation, the strategy was changed to a conservative strategy, which was consistent with the findings of Pinho et al. [73]. The SLA index increased with the accrue of crown canopy and nutrient utilization, and the SLA index of dominant species decreased with the complexity of the community structure during restoration [87]. Our research also confirmed that the SLA of *Q*. *wutaishanica* showed similar changes in community diversity and soil NH_4_^+^-N. Moreover, SLA is affected by both LT and LDMC indices [88]. In our study, the LT and LDMC indices decreased at the beginning and then increased with restoration time, and the change in LT was greater. As the LT index was positively correlated with the utilization efficiency of light resources [89], *Q*. *wutaishanica* gradually occupied the canopy position as the dominant species in community restoration, which improved the utilization efficiency of light resources by the leaves [90].

The changes in plant functional traits of the dominant species are driven by carbon and other nutrient cycles [91]. The higher the leaf nitrogen and phosphorus contents, the greater the photosynthetic rate, while the growth rate and resource competitiveness increased accordingly [92]. We found that leaf TN content gradually increased and tended to be stable with the prolonging of the restoration period, which further supports the conclusion that TN content increases with increasing stand age [93]. The leaf TP content decreased gradually with restoration, which was caused by various nutrient utilization strategies of different plant types and explained the functional redundancy of soil AP at different restoration stages [94].

Plant-soil interactions are an important determinant of vegetation restoration [95, 96]. We found a strong correlation between soil TC, FDis, and RaoQ after 10 years of restoration, and the limiting effect gradually decreased, indicating that the resource utilization strategy of *Q*. *wutaishanica* changed during community restoration. It has been reported that the reduction in the utilization efficiency of light resources by plants leads to the weakening of the nutrient cycle restriction [73]. They also showed that stand age is an important driver of soil carbon input [28]. At the early stage of restoration, plants have a strong demand for nutrients, but less input of leaf litter makes it difficult to achieve effective carbon sequestration [91]. On the other hand, with community restoration, soil carbon input increased, which increased soil functional redundancy and improved geobiochemical circulation [97]. In addition, the effect of higher species richness in the initial restoration stages of plant communities on plant seed quality was also influenced by the supply of soil resources. As restoration proceeds, competition among plant consumers increases intraspecific community height and decreases interspecific SLA [69, 70]. Our study further confirmed that plant leaf nitrogen content had an important effect on species richness from the late stage of community restoration, which was consistent with previous findings that also reported that changes in plant functional traits affected species richness [98].

## 5 Conclusion

*Q*. *wutaishanica* is the dominant tree species in natural ecosystem restoration of temperate forests in China, which plays an active role in maintaining ecological balance. However, little is known about how ecosystem versatility develops during the restoration of forest ecosystems dominated by *Q*. *wutaishanica*. In this study, we investigated the species composition of *the Q*. *wutaishanica* community, soil nutrients, and their functional traits at various restoration stages, and comprehensively analyzed the correlations among them. At the early stage of restoration (10 years of restoration), there were more shrubs in *Q*. *wutaishanica* community, while had a larger niche width. In the middle of restoration (30 years of restoration), shannon and richness indexes were the largest, while soil total carbon, total phosphorus, ammonium nitrogen and leaf shape index, chlorophyll content of *Q*. *wutaishanica* leaves were the highest. Moreover, the correlation between leaf and soil nutrients was the strongest. At the later stage of restoration (40 years of restoration), soil available nitrogen, available phosphorus content and leaf thickness were the largest. However, the restriction of various soil nutrients was reduced. Our study highlighted the effectiveness of soil resource availability in plant communities during restoration, reduced competition for light among plants, and altered species richness. Furthermore, changes in the interrelationship between plant community composition and leaf functional traits of the dominant species responded positively to community restoration. These results further deepen our understanding of forest management and restoration of forest communities. In the future, it is necessary to comprehensively consider the influence of various factors on forest community restoration.

## Supporting information

S1 Data (XLSX)

## References

[1] Deng L.; Wang K.B.; Chen M.L.; Shangguan Z.P.; Sweeney S. Soil organic carbon storage capacity positively related to forest succession on the Loess Plateau, China. *Catena* 2013, 110, 1–7. doi: 10.1016/j.catena.2013.06.016

[2] Zhang Y.; Peng C.H.; Li W.Z.; Tian L.X.; Zhu Q., Chen H., et al. Multiple afforestation programs accelerate the greenness in the ‘Three North’ region of China from 1982 to 2013. *Ecol Indic*. 2016, 61, 404–412. doi: 10.1016/j.ecolind.2015.09.041

[3] Yang H. China’s Natural Forest Protection Program: Progress and impacts. *Forest Chron*. 2017, 93, 113–117. doi: 10.5558/tfc2017-017

[4] Xiang W.H.; Zhou J.; Ouyang S.; Zhang S.L.; Lei P.F.; Li J.X.; et al. Species-specific and general allometric equations for estimating tree biomass components of subtropical forests in southern China. *Eur J Forest Res*. 2016, 135, 963–979. doi: 10.1007/s10342-016-0987-2

[5] Zeng Y.L.; Fang X.; Xiang W.H.; Deng X.W.; Peng C.H. Stoichiometric and nutrient resorption characteristics of dominant tree species in subtropical Chinese forests. *Ecol Evol*. 2017, 7, 11033–11043. doi: 10.1002/ece3.3527 29299279 PMC5743644

[6] Zou N.G.; Luo W.X. Silviculture practice in Loess Plateau of China (in Chinese). Chinese Forestry Press, Beijing. 1997, 576908. doi: 10.3389/ffgc.2021.576908

[7] Isbell F.; Craven D.; Connolly J.; Loreau M.; Schmid B.; Beierkuhnlein C.; et al. Biodiversity increases the resistance of ecosystem productivity to climate extremes. *Nature* 2015, 526, 574–577. doi: 10.1038/nature15374 26466564

[8] Bergeron Y. Species and stand dynamics in the mixed woods of Quebec’s southern boreal forest. *Ecology*. 2000, 81, 1500–1516. 10.1890/0012-9658(2000)081[1500:SASDIT]2.0.CO;2.

[9] Iida Y.; Poorter L.; Sterck F.; Kassim A.R.; Potts M.D.; Kubo T.; et al. Linking size-dependent growth and mortality with architectural traits across 145 co-occurring tropical tree species. *Ecology*. 2014, 95, 353–363. doi: 10.1890/11-2173.1 24669729

[10] Slik J.W.F.; Bernard C.S.; Breman F.C.; Van Beek M.; Salim A.; Sheil D. Wood density as a conservation tool: Quantification of disturbance and identification of conservation-priority areas in tropical forests. *Conserv Biol*. 2008, 22, 1299–1308. doi: 10.1111/j.1523-1739.2008.00986.x 18637916

[11] Laurance W.; Nascimento H.; Laurance S.; Andrade A.; Ribeiro J.E.L.S.; Giraldo J.P.; et al. Rapid decay of tree-community composition in Amazonian forest fragments. *P Natl Acad SCI USA* 2006, 103, 19010–19014. doi: 10.1073/pnas.0609048103 17148598 PMC1682011

[12] Hipp A.L.; Larkin D.J.; Barak R.S.; Bowles M.L.; Cadotte M.W.; Jacobi S.K.; et al. Phylogeny in the service of ecological restoration. Am. *J Bot*. 2015, 102, 647–648. doi: 10.3732/ajb.1500119 26022478

[13] Barber N.A.; Jones H.P.; Duvall M.R.; Wysocki W.P.; Hansen M.J.; Gibson D.J. Phylogenetic diversity is maintained despite richness losses over time in restored tallgrass prairie plant communities. *J Appl Eco*. 2017, 54, 137–144. doi: 10.1111/1365-2664.12639

[14] Loreau M.; Naeem S.; Inchausti P.; Bengtsson J.; Grime J.P.; Hector A.; et al. Biodiversity and ecosystem functioning: current knowledge and future challenges. *Science* 2001, 294, 804–808. doi: 10.1126/science.1064088 11679658

[15] Cardinale B. J.; Srivastava D. S.; Duffy J. E.; Wright J. P.; Downing A. L.; Sankaran M.; et al. Effects of biodiversity on the functioning of trophic groups and ecosystems. *Nature* 2006, 443, 989–992. doi: 10.1038/nature05202 17066035

[16] Hedwall P.O.; Bergh J.; Brunet J. Phosphorus and nitrogen co-limitation of forest ground vegetation under elevated anthropogenic nitrogen deposition. *Oecologia*. 2017, 185, 317–326. doi: 10.1007/s00442-017-3945-x 28884383 PMC5617880

[17] van Breugel M.; van Breugel P.; Jansen P.A.P.A.; Martínez-Ramos M.; Bongers F. The relative importance of above- versus belowground competition for tree growth during early succession of a tropical moist forest. *Plant Ecol*. 2012, 213, 25–34. doi: 10.1007/s11258-011-0003-3

[18] Zethof J.H.T.; Cammeraat E.L.H.; Nadal-Romero E. The enhancing effect of afforestation over secondary succession on soil quality under semiarid climate conditions. *Sci Total Environ*. 2019, 652, 1090–1101. doi: 10.1016/j.scitotenv.2018.10.235 30586796

[19] Melvin K.K.K.; Japar S.B.; Osumanu H.A.; Nik M.A.M.; Roland K.J.H.; Silvester J. Comparison of carbon and selected macronutrients in forest-floor litter of rehabilitated and secondary forests. *Am J Appl Sci*. 2011, 8, 967. doi: 10.3844/ajassp.2011.967.972

[20] Fayolle A.; Engelbrecht B.; Freycon V.; Mortier F.; Swaine M.; Réjou-Méchain M.; et al. Geological substrates shape tree species and trait distributions in African moist forests. *PloS One* 2012, 7, e42381. doi: 10.1371/journal.pone.0042381 22905127 PMC3419707

[21] Lucas-Borja M.E.; Hedo J.; Cerda A.; Candel-Perez D.; Vinegla B. Unravelling the importance of forest age stand and forest structure driving microbiological soil properties, enzymatic activities and soil nutrients content in Mediterranean Spanish black pine (Pinus nigra Ar. ssp salzmannii) Forest. *Sci Total Environ*. 2016, 562, 145–154. doi: 10.1016/j.scitotenv.2016.03.160 27099995

[22] Zhang W.; Zhao J.; Pan F.J.; Li D.J.; Chen H.S.; Wang K.L. Changes in nitrogen and phosphorus limitation during secondary succession in a karst region in southwest China. *Plant Soil*. 2015, 391, 77–91. doi: 10.1007/s11104-015-2406-8

[23] Rejou-Mechain M.; Flores O.; Pelissier R.; Fayolle A.; Fauvet N.; Gourlet-Fleury S. Tropical tree assembly depends on the interactions between successional and soil filtering processes. *Global Ecol Biogeogr*. 2014, 23, 1440–1449. doi: 10.1111/geb.12222

[24] Villa P.M.; Martins S.V.; de Oliveira Neto S.N.; Rodrigues A.C.; Safar N.V.H.; Monsanto L.D.; et al. Woody species diversity as an indicator of the forest recovery after shifting cultivation disturbance in the northern Amazon. *Ecol Ind*. 2018, 95, 687–694. doi: 10.1016/j.ecolind.2018.08.005

[25] Reich P.B. The world-wide ‘fast-slow’ plant economics spectrum: a traits mani festo. *J Ecol*. 2014, 102, 275–301. doi: 10.1111/1365-2745.12211

[26] Prieto I.; Querejeta J.I.; Segrestin J.; Volaire F.; Roumet C. Leaf carbon and oxygen isotopes are coordinated with the leaf economics spectrum in Mediterranean rangeland species. *Funct Ecol*. 2018, 32, 612–625. doi: 10.1111/1365-2435.13025

[27] Boukili V.K.; Chazdon R.L. Environmental filtering, local site factors and landscape context drive changes in functional trait composition during tropical forest succession. *Perspect Plant Ecol*. 2017, 24, 37–47. doi: 10.1016/j.ppees.2016.11.003

[28] Lohbeck M.; Poorter L.; Lebrija-Trejos E.; Martínez-Ramos M.; Meave J.A.; Paz H.; et al. Successional changes in functional composition contrast for dry and wettropical forest. *Ecology* 2013, 94, 1211–1216. doi: 10.1890/12-1850.1 23923479

[29] Wright I.J.; Reich P.B.; Westoby M. Strategy shifts in leaf physiology, structure and nutrient content between species of high-and low-rainfall and high-and low nutrient habitats. *Funct Ecol*. 2001, 15, 423–434. doi: 10.1046/j.0269-8463.2001.00542.x

[30] Meiners S.J.; Cadotte M.W.; Fridley J.D.; Pickett S.T.; Walker L.R. Is successional research nearing its climax? New approaches for understanding dynamic communities. *Funct Ecol*. 2015, 29, 154–164. doi: 10.1111/1365-2435.12391

[31] Mensah S.; Glèlè Kakaï R.; Seifert T. Patterns of biomass allocation between foliage and woody structure: the effects of tree size and specific functional traits. *Ann For Res*. 2016, 59, 49–60. doi: 10.15287/afr.2016.458

[32] Li G.; Liu C.; Liu Y.; Yang J.; Zhang X.S.; Guo K. Effects of climate, disturbance and soil factors on the potential distribution of Liaotung oak (*Quercus wutaishanica* Mayr) in China. *Ecol Res*. 2012, 27, 427–436. doi: 10.1007/s11284-011-0914-4

[33] Yin Q.L.; Tian T.T.; Han X.H.; Ju J.S.; Chai Y.F.; Mo J.; et al. The relationships between biomass allocation and plant functional trait. *Ecol Indic*. 2019, 102, 302–308. doi: 10.1016/j.ecolind.2019.02.047

[34] Bai X.J.; Wang B.R.; An S.S.; Zeng Q.C.; Zhang H.X. Response of forest species to C:N:P in the plant-litter-soil system and stoichiometric homeostasis of plant tissues during afforestation on the Loess Plateau, China. *Catena*. 2019, 183, 104186. doi: 10.1016/j.catena.2019.104186

[35] Cheng X.; Ping T.; Li Z.; Tian W.; Hairong H.; Epstein H.E. Effects of environmental factors on plant functional traits across different plant life forms in a temperate forest ecosystem. *New Forest*. 2021, 53, 1–8. doi: 10.1007/s11056-021-09847-0

[36] Wang Z.; Zheng F. Impact of vegetation succession on leaf-litter-soil C:N:P stoichiometry and their intrinsic relationship in the Ziwuling Area of China’s Loess Plateau. *J Forestry Res*. 2021, 32, 697–711. doi: 10.1007/s11676-020-01149-z

[37] Zhang Z.H.; Guo J. B.; Wang Y.H.; Wang X. Population structure and spatial distribution pattern of *Quercus wutaishanica* in Liupan Mountains. J Zhejiang A & F University. 2021, 38, 1091–1099. (in Chinese).

[38] Cheng J.M.; Zhu R.B.; Wang S.G. A photographic guide to plants of Liupanshan, Beijing Science Press, 2020.

[39] Shannon C.E.; Weaver W. The Mathematical Theory of Communication. Univ. of Illinois Press. 1949, 27, 1–117. https://www.press.uillinois.edu/books/?id=p725487.

[40] Pielou E.C. The measurement of diversity in different types of biological collections. *J*. *Theor*. 1966, 13, 131–144. doi: 10.1016/0022-5193(66)90013-0

[41] Zhang L.J.; Yue M.; Zhang Y.D.; Gu F.X.; Pan X.L.; Zhao G.F. Characteristics of plant community species diversity of oasis desert ecotone in Fukang, Xinjiang. *Sci*. *Geol*. *Sin*. 2003, 23, 329–334. http://geoscien.neigae.ac.cn/EN/10.13249/j.cnki.sgs.2003.03.329.

[42] Levins R. *Evolution In Changing Environments*: *Some Theoretical Explorations*; Princeton: Princeton UP, USA, 1968. doi: 10.2307/j.ctvx5wbbh

[43] Hurlbert S.H. The measurement of niche overlap and some relatives. *Ecology* 1978, 59, 67–77. doi: 10.2307/1936632

[44] Bao S. D. Soil and Agricultural Chemistry Analysis. China Agriculture Publication. 2000.

[45] Wu Y.; Chen W.; Li Q.; Guo Z.; Zhao Z.; Zhai J.; et al. Ecoenzymatic stoichiometry and nutrient limitation under a natural secondary succession of vegetation on the Loess Plateau, China. *Land Degrad Dev*. 2021, 32, 399–409. doi: 10.1002/ldr.3723

[46] Han T.; Ren H.; Wang J.; Lu H.; Song G.; Chazdon R.L. Variations of leaf eco-physiological traits in relation to environmental factors during forest succession. *Ecol*. *Indic*. 2020, 117, 106511. doi: 10.1016/j.ecolind.2020.106511

[47] Dong M. Survey, observation and analysis of terrestrial biocommunities; Standards Press of China, Beijing, China, 1997.

[48] Lichtenthaler H.K.; Gitelson A.A.; Lang M. Non-destructive determination of chlorophyll content of leaves of a green and an aurea mutant of tobacco by reflectance measurements. *J Plant Physiol*. 1996, 148, 483–493. doi: 10.1016/S0176-1617(96)80283-5

[49] Lozupone C.; Lladser M.E.; Knights D.; Stombaugh J.; Knight R. UniFrac: an effective distance metric for microbial community comparison. *ISME J*. 2011, 5, 169–172. doi: 10.1038/ismej.2010.133 20827291 PMC3105689

[50] Long J.D.; Turner D. Applied R in the Classroom. *Aust Econ Rev*. 2020, 53, 139–157. doi: 10.1111/1467-8462.12362

[51] Zhang J.T.; Chen T. Effects of mixed Hippophae rhamnoides on community and soil in planted forests in the Eastern Loess Plateau, China. *Ecol Eng*. 2007, 31, 115–121. doi: 10.1016/j.ecoleng.2007.06.003

[52] Craven D.; Hall J.S.; Berlyn G.P.; Ashton M.S.; van Breugel M. Changing gears during succession: Shifting functional strategies in young tropical secondary forests. *Oecologia*. 2015, 179, 293–305. doi: 10.1007/s00442-015-3339-x 25990298

[53] Abbas S.; Nichol J.E.; Zhang J.L.; Fischer G.A. The accumulation of species and recovery of species composition along a 70 year succession in a tropical secondary forest. *Ecol Indic*. 2019, 106, 105524. doi: 10.1016/j.ecolind.2019.105524

[54] Tabarelli M.; Lopes A.V.; Peres C.A. Edge-effects drive tropical forest fragments towards an early-successional system. *Biotropica*. 2008, 40, 657–661. doi: 10.1111/j.1744-7429.2008.00454.x

[55] Rivas-Alonso E.; Martinez-Garza C.; de la Pena-Domene M.; Mendez-Toribio M. Large trees in restored tropical rainforest. *Forest Ecol Manag*. 2021, 498, 119563. doi: 10.1016/j.foreco.2021.119563

[56] Abbas S.; Nichol J.E.; Zhang J.L.; Fischer G.A.; Wong M.S.; Irteza S.M. Spatial and environmental constraints on natural forest regeneration in the degraded landscape of Hong Kong. *Sci Total Environ*. 2021, 752, 141760. doi: 10.1016/j.scitotenv.2020.141760 32890826

[57] Holl K.D.; Stout V.M.; Reid J.L.; Zahawi R.A. Testing heterogeneity-diversity relationships in tropical forest restoration. *Oecologia*. 2013, 173, 569–578. doi: 10.1007/s00442-013-2632-9 23525802

[58] Garcia L.C.; Hobbs R.J.; Ribeiro D.B.; Tamashiro J.Y.; Santos F.A.M.; Rodrigues R.R. Restoration over time: is it possible to restore trees and non-trees in high-diversity forests? *Appl Veg Sci*. 2016, 19, 655–666. doi: 10.1111/avsc.12264

[59] Gandolfi S.; Joly C.A.; Rodrigues R.R. Permeability-impermeability: canopy trees as biodiversity filters. *Sci Agr*. 2007, 64, 433–438. doi: 10.1590/S0103-90162007000400015

[60] Sheil D.; Burslem D.F.R.P. Disturbing hypotheses in tropical forests. *Trends Ecol Evol*. 2003, 18, 18–26. doi: 10.1016/S0169-5347(02)00005-8

[61] Catford J.A.; Daehler C.C.; Murphy H.T.; Sheppard A.W.; Hardesty B.D.; Westcott D.A.; et al. The intermediate disturbance hypothesis and plant invasions: Implications for species richness and management. *Perspect Plant Ecol*. 2012, 14, 231–241. doi: 10.1016/j.ppees.2011.12.002

[62] Lucas-Borja M.E.; Candel-Pérez D.; Morote F.A.G.; Onkelinx T.; Tíscar P.A.; Balandier P. Pinus nigra Arn. Ssp. salzmannii seedling recruitment is affected by stand basal area, shrub cover and climate interactions. *Ann Forest Sci*. 2016, 73, 649–656. doi: 10.1007/s13595-016-0550-9

[63] Lasky J.R.; Uriarte M.; Boukili V.K.; Erickson D.L.; John Kress W.; Chazdon R.L. The relationship between tree biodiversity and biomass dynamics changes with tropical forest succession. *Ecol Lett*. 2014, 17, 1158–1167. doi: 10.1111/ele.12322 24986005

[64] Chua S.C.; Ramage B.S.; Ngo K.M.; Potts M.D.; Lum S.K.Y. Slow recovery of a secondary tropical forest in Southeast Asia. *Forest Ecol Manag*. 2013, 308, 153–160. doi: 10.1016/j.foreco.2013.07.053

[65] Shoo L.P.; Freebody K.; Kanowaski J.; Catterrall C.P. Slow recovery of tropical old-field rainforest regrowth and the value and limitations of active restoration. *Conserv Biol*. 2016, 30, 121–132. doi: 10.1111/cobi.12606 26310383

[66] Muller S.C.; Bergamin R.S.; Bordin K.M.; Klipel J.; Rosenfield M.F. Canopy Leaf Traits, Basal Area, and Age Predict Functional Patterns of Regenerating Communities in Secondary Subtropical Forest. *Front Plant Sci*. 2021, 4, 572864. doi: 10.3389/ffgc.2021.572864

[67] Chang X.Y.; Zhou T.; Peng S.L. Change in community phylogenetic structure of tropical forest along the southern coast of China during restoration. *Ecospere*. 2015, 6, 1–13. doi: 10.1890/ES15-00118.1

[68] Arroyo-Rodríguez V.; Melo F. P.; Martínez-Ramos M.; Bongers F.; Chazdon R. L.; Meave J. A.; et al. Multiple successional pathways in human-modified tropical landscapes: New insights from forest succession, forest fragmentation and landscape ecology research. *Biol Rev*. 2017, 92, 326–340. doi: 10.1111/brv.12231 26537849

[69] Wilfahrt P.A.; Halliday F.W.; Heckman R.W. Initial richness, consumer pressure and soil resources jointly affect plant diversity and resource strategies during a successional field experiment. *J Ecol*. 2020, 108, 2352–2365. doi: 10.1111/1365-2745.13396

[70] Heckman R.W; Halliday F.W.; Wilfahrt P.A. Nutrients and consumers impact tree colonization differently from performance in a successional old field. *Oecologia*. 2022, 198, 219–227. https://doi.org/ 10.1007/s00442-021-05096-2. 35079868 10.1007/s00442-021-05096-2

[71] Letcher S.G. Phylogenetic structure of angiosperm communities during tropical forest succession. *Proc Roy Soc B-Biol Sci*. 2010, 277, 97–104. https://www.jstor.org/stable/40506093. doi: 10.1098/rspb.2009.0865 19801375 PMC2842617

[72] Proches S. Wilson J.R.U. Richardson D.M. Rejmanek M. Searching for phylogenetic pattern in biological invasions. *Global Ecol Biogeogr*. 2008, 17, 5–10. doi: 10.1111/j.1466-8238.2007.00333.x

[73] Pinho B.X.; de Melo F.P.L.; Arroyo-Rodriguez V.; Pierce S.; Lohbeck M.; Tabarelli M. Soil-mediated filtering organizes tree assemblages in regenerating tropical forests. *J Ecol*. 2018, 106, 137–147. doi: 10.1111/1365-2745.12843

[74] Brenes-Arguedas T.; Ríos M.; Rivas-Torres G.; Blundo C.; Coley P.D.; Kursar T.A. The effect of soil on the growth performance of tropical species with contrasting distributions. *Oikos*. 2008, 117, 1453–1460. doi: 10.1111/j.0030-1299.2008.16903.x

[75] Lucas-Borja M.E.; Delgado-Baquerizo M. Plant diversity and soil stoichiometry regulates the changes in multifunctionality during pine temperate forest secondary succession. *Sci Total Environ*. 2019, 697, 134204. doi: 10.1016/j.scitotenv.2019.134204 31491638

[76] van Breugel M.; Craven D.; Lai H.R.; Baillon M.; Turner B.L.; Hall J.S. Soil nutrients and dispersal limitation shape compositional variation in secondary tropical forests across multiple scales. *J Ecol*. 2019, 107, 566–581. doi: 10.1111/1365-2745.13126

[77] Wright I.J.; Reich P.B.; Cornelissen J.H.C.; Falster D.S.; Garnier E.; Hikosaka K.; et al. Assessing the generality of global leaf trait relationships. *New Phytol*. 2005, 166, 485–496. doi: 10.1111/j.1469-8137.2005.01349.x 15819912

[78] Ordonez J.C.; van Bodegom P.M.; Witte J. P. M.; Wright I.J.; Reich P.B.; Aerts R. A global study of relationships between leaf traits, climate and soil measures of nutrient fertility. *Global Ecol Biogeogr*. 2009, 18, 137–149. doi: 10.1111/j.1466-8238.2008.00441.x

[79] Reed S.C.; Cleveland C.C.; Townsend A.R. Functional ecology of free-living nitrogen fixation: a contemporary perspective. *Annu Rev Ecol Evol S*. 2011, 42, 489–512. doi: 10.1146/annurev-ecolsys-102710-145034

[80] Wang M.; Xu J.S.; Chai Y.F.; Guo Y.X.; Liu X.; Yue M. Differentiation of environmental conditions promotes variation of two *Quercus wutaishanica* community assembly patterns. *Forests*. 2020, 11. 43. doi: 10.3390/f11010043

[81] Bauhus J.; Pare D. Effects of tree species, stand age and soil type on soil microbial biomass and its activity in a southern boreal forest. *Soil Biol*. *Biochem*. 1998, 30, 1077–1089. doi: 10.1016/S0038-0717(97)00213-7

[82] Díaz S.; Kattge J.; Cornelissen J.H.C.; Wright I.J.; Lavorel S.; Dray S.; et al. The global spectrum of plant form and function. *Nature*. 2016, 529, 167–171. doi: 10.1038/nature16489 26700811

[83] Cadotte M.W; Tucker C.M. Should environmental filtering be abandoned? *Trends Ecol Evol*. 2017, 32, 429–437. doi: 10.1016/j.tree.2017.03.004 28363350

[84] Pierce S.; Negreiros D.; Cerabolini B.E.L.; Kattge J.; Díaz S.; Kleyer M.; et al. A global method for calculating plant CSR ecological strategies applied across biomes world-wide. *Funct Ecol*. 2017, 31, 444–457. doi: 10.1111/1365-2435.12722

[85] Thom D.; Taylor A.R.; Seidl R.; Thuiller W.; Wang J.J.; Robideau M.; et al. Forest structure, not climate, is the primary driver of functional diversity in northeastern North America. *Sci Total Environ*. 2021, 762, 143070. doi: 10.1016/j.scitotenv.2020.143070 33127131 PMC7612768

[86] Craven D.; Hall J.S.; Berlyn G.P.; Ashton M.S.; van Breugel M. Environmental filtering limits functional diversity during succession in a seasonally wet tropical secondary forest. *J Veg Sci*. 2018, 29, 511–520. doi: 10.1111/jvs.12632

[87] Muscarella R.; Lohbeck M.; Martínez-Ramos M.; Poorter L.; Rodríguez-Velázquez J.E.; van Breugel M.; et al. Demographic drivers of functional composition dynamics. *Ecology* 2017, 98, 2743–2750. doi: 10.1002/ecy.1990 28833040

[88] Hodgson J.G.; Montserrat-Martí G.; Charles M.; Jones G.; Wilson P.; Shipley B.; et al. Is leaf dry matter content a better predictor of soil fertility than specific leaf area? *Ann Bot*. 2011, 108, 1337–1345. doi: 10.1093/aob/mcr225 21948627 PMC3197453

[89] Witkowski E.T.F.; Lamont B.B. Leaf specific mass confounds leaf density and thickness. *Oecologia*. 1991, 88, 486–493. doi: 10.1007/BF00317710 28312617

[90] Dengler N.G. Comparative histological basis of sun and shade leaf dimorphism in Helianthus annuus. *Can*. *J*. *Bot*. 1980, 58, 717–730. doi: 10.1139/b80-092

[91] Teixeira H.M.; Cardoso I.M.; Bianchi F.J.J.A.; Silva A.D.; Jamme D.; Pena-Claros M. Linking vegetation and soil functions during secondary forest succession in the Atlantic forest. *Forest Ecol Manag*. 2020, 457, 117696. doi: 10.1016/j.foreco.2019.117696

[92] Niklas K.J.; Cobb E.D. N, P, and C stoichiometry of Eranthishyemalis (Ranunculaceae) and the allometry of plant growth. *Am J Bot*. 2005, 92, 1256–1263. doi: 10.2307/4126089 21646146

[93] Warring B.; Cardoso F.C.G.; Marques M.C.M.; and Varassin I.G. Functional diversity of reproductive traits increases across succession in the Atlantic forest. *Rodriguesia*. 2016, 67, 321–333. doi: 10.1590/2175-7860201667204

[94] Schreeg L.A.; Santiago L.S.; Wright S.J.; Turner B.L. Stem, root, and older leaf N: P ratios are more responsive indicators of soil nutrient availability than new foliage. *Ecology* 2014, 95, 2062–2068. doi: 10.1890/13-1671.1 25230458

[95] Meli P.; Holl K.D.; Rey Benayas J.M.; Jones H.P.; Jones P.C.; Montoya D.; et al. A global review of past land use, climate, and active vs. passive restoration effects on forest recovery. *PLoS One* 2017, 12, e0171368. doi: 10.1371/journal.pone.0171368 28158256 PMC5291368

[96] Suganuma M.S.; Torezan J.M.D.; Durigan G. Environment and landscape rather than planting design are the drivers of success in long-term restoration of riparian Atlantic forest. *Appl Veg Sci*. 2018, 21, 76–84. doi: 10.1111/avsc.12341

[97] Poorter L.; Bongers F.; Aide T.M.; Almeyda Zambrano A.M.; Balvanera P.; Becknell J.M.; et al. Biomass resilience of Neotropical secondary forests. *Nature* 2016, 530, 211–214. doi: 10.1038/nature16512 26840632

[98] Finegan B.; Pena-Claros M.; de Oliveira A.; Ascarrunz N.; Bret-Harte M.S.; Carreno-Rocabado G.; et al. Does functional trait diversity predict above-ground biomass and productivity of tropical forests? Testing three alternative hypotheses. *J Ecol*. 2014, 103, 191–201. doi: 10.1111/1365-2745.12346

